# How the structure of Wikipedia articles influences user navigation

**DOI:** 10.1080/13614568.2016.1179798

**Published:** 2016-05-12

**Authors:** Daniel Lamprecht, Kristina Lerman, Denis Helic, Markus Strohmaier

**Affiliations:** ^a^ Knowledge Technologies Institute, Graz University of Technology, Graz, Austria; ^b^ Information Sciences Institute, University of Southern California, Los Angeles, CA, USA; ^c^ Department of Computer Science, University of Koblenz-Landau, Mainz, Germany; ^d^ GESIS – Leibniz Institute for the Social Sciences, Cologne, Germany

**Keywords:** Wikipedia, navigation, article structure, generality

## Abstract

In this work we study how people navigate the information network of Wikipedia and investigate (i) free-form navigation by studying all clicks within the English Wikipedia over an entire month and (ii) goal-directed Wikipedia navigation by analyzing wikigames, where users are challenged to retrieve articles by following links. To study how the organization of Wikipedia articles in terms of layout and links affects navigation behavior, we first investigate the characteristics of the structural organization and of hyperlinks in Wikipedia and then evaluate link selection models based on article structure and other potential influences in navigation, such as the generality of an article's topic. In free-form Wikipedia navigation, covering all Wikipedia usage scenarios, we find that click choices can be best modeled by a bias towards article structure, such as a tendency to click links located in the lead section. For the goal-directed navigation of wikigames, our findings confirm the zoom-out and the homing-in phases identified by previous work, where users are guided by generality at first and textual similarity to the target later. However, our interpretation of the link selection models accentuates that article structure is the best explanation for the navigation paths in all except these initial and final stages. Overall, we find evidence that users more frequently click on links that are located close to the top of an article. The structure of Wikipedia articles, which places links to more general concepts near the top, supports navigation by allowing users to quickly find the better-connected articles that facilitate navigation. Our results highlight the importance of article structure and link position in Wikipedia navigation and suggest that better organization of information can help make information networks more navigable.

## 1. Introduction

Much of human knowledge and expertise resides in networks, such as the World Wide Web, Wikipedia, scientific citation networks, and, increasingly, user-generated content on social media. Successfully finding relevant information in these networks, even as they become larger and more complex, is key to our continued ability to innovate, grow, and prosper. While search engines have drastically facilitated information-seeking, not every information need is directly satisfiable. In situations when a query cannot be expressed in an explicit fashion, navigation and exploration are necessarily the information retrieval techniques of choice. Information needs are generally dynamic and evolving (e.g. as modeled by Berrypicking Bates, 1989 or Information Scent Chi et al., 2001), and knowledge gained during the navigation process can put information in context and help with decision-making (Marchionini, 2006). Some users prefer navigation over search even when they know what they are looking for Teevan et al. (2004). For a large encyclopedia such as Wikipedia, possible navigation scenarios generally can span a large range, from goal-directed navigation to following a link to learn more about a certain concept, to explorative search and many more.

*Problem*. Many questions about information networks, and how people navigate them to find relevant information, have not yet been fully answered. For example, how should webpages be structured to facilitate navigation and searchability? How does an individual's familiarity with the knowledge contained in the network influence navigation? Answering question such as these will help to create efficient navigation structures in massive information networks. Generally, more attention is paid to items that are displayed at the top of the screen or the top of a list of items, even when no ranking is present (Payne, 1951). For webpages, users scan the pages in an f-shaped pattern (Nielsen, 2006) and dedicate more attention to the top and left (Buscher, Cutrell, and Morris, 2009).

On Wikipedia, articles are subject to a common page organization. For example, the first section usually introduces the article in more general and more broadly accessible terms, and the infobox summarizes the main facts. We therefore hypothesize that the accessibility of the topmost section and this clear organization helps users to more easily find relevant links and to successfully navigate Wikipedia. Hence, we study how the organization of Wikipedia articles in terms of sections, infoboxes and link positions affects navigation in Wikipedia. Previous work has shown both semantic and structural knowledge to influence Web navigation (Juvina and van Oostendorp, 2008). For goal-directed Wikipedia navigation, users have been found to select links based on both semantic similarity and overlap of link titles (Salmerón, Cerdán, and Naumann, 2015). In this work, we compare the influence of textual similarity of articles to the influence of structural elements. Specifically, we examine the following research question:

*Research question*: To what extent does article structure affect Wikipedia navigation?

*Approach*. We address this questions by studying how people use Wikipedia to find information. Our approach is two-fold:

- We analyze an entire month of all clicks within the English Wikipedia. This permits us to gain insight into unrestricted free-form clicking behavior on Wikipedia, covering all usage scenarios.
- We analyze wikigames, which challenge people to navigate from a source Wikipedia article to a given target article solely by using the existing links in the text. Wikigames allow us to inspect goal-directed Wikipedia navigation from the perspective of very focused navigation set up as a game, for which we have access to very detailed log files and can investigate clicking behavior step by step.

**Figure 1.**
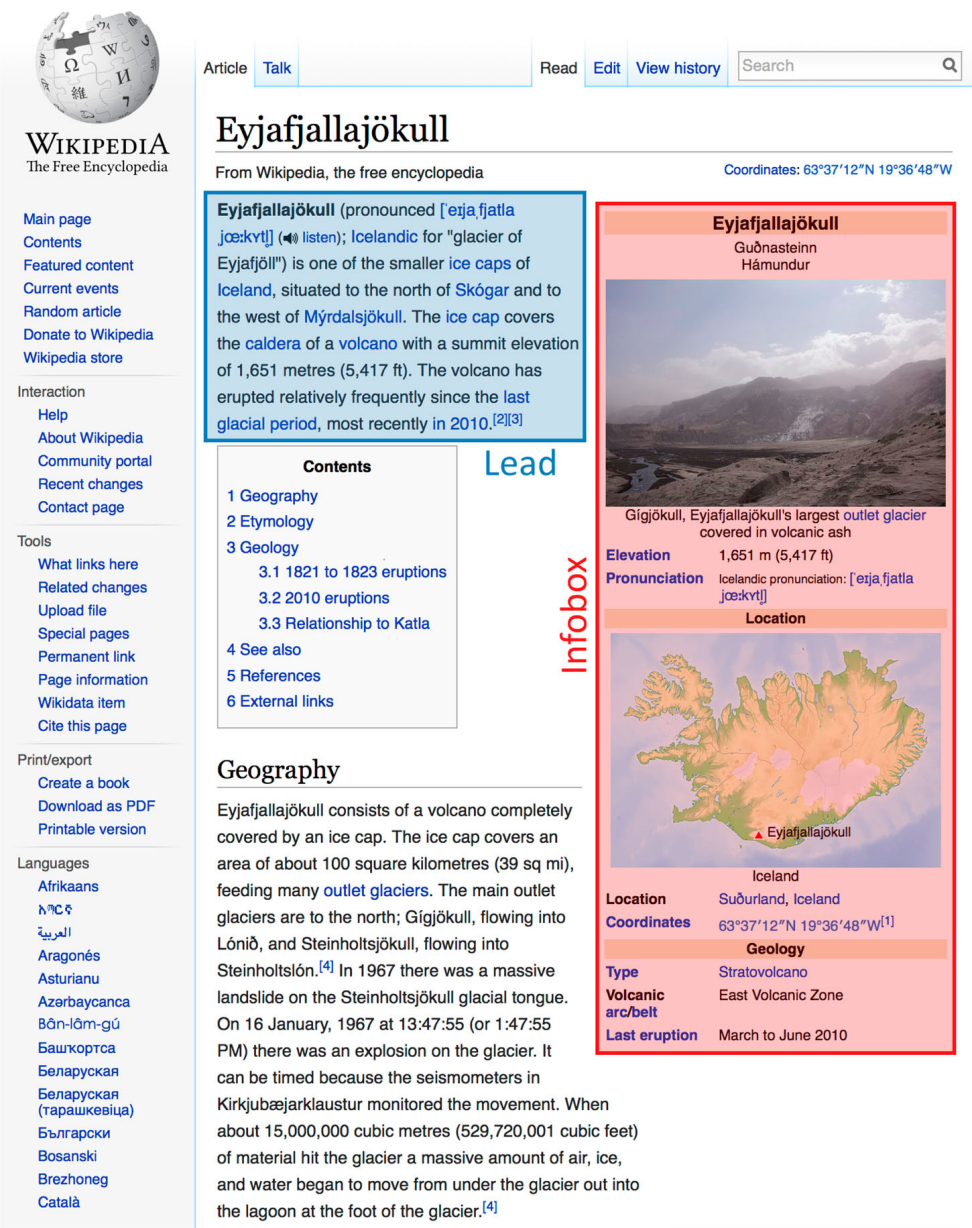
*Example of the structure of a Wikipedia article.* The image shows a Wikipedia article with the lead section (blue shading) and the infobox (red shading), followed by the table of contents and the start of the main content. In this paper we show that users focus their attention on the lead sections and the infoboxes.

We use these two datasets to study the structure of Wikipedia article in terms of lead sections and infoboxes (see Figure 1 for an example). We first investigate the characteristics of these sections and of hyperlinks in Wikipedia and then analyze potential influences on navigation by simulating link selection models based on a range of influence factors. Finally, we consider the detailed logs of goal-directed wikigames and study link selection behavior step by step.

*Contributions*. Our results suggest that article structure has a strong influence on navigation. We find evidence that a large share of user clicks are to links in the lead section or an infobox. For free-form Wikipedia navigation, navigation decisions can be best explained by a bias towards the article structure, favoring links located near the top of the article. For the goal-directed navigation of wikigames, our findings confirm the zoom-out and the homing-in phases identified therein by previous work (West and Leskovec, 2012b), where users are guided by generality at first and textual similarity to the target towards the end. However, the outcomes of the link selection models accentuate that article structure is the best explanation for wikigames in all except these initial and final stages. Our results highlight the importance of article structure and link position in Wikipedia navigation and suggest that better organization of information can help make information networks more navigable.

## 2. Related work

### 2.1. Navigation in social networks

Research on navigation in networks was brought into being in the 1960s by Stanley Milgram's influential letter forwarding experiments. These experiments established that participants were able to find short chains between unrelated individuals in the social network of the entire United States (Milgram, 1967) with a decentralized search approach (i.e. the search problem was forwarded with a letter and not controlled by a centralized instance).

In the 1990s, Watts and Strogatz demonstrated that many social, technological and biological networks exhibited the *small-world property* (high clustering and a small diameter), which ensured that most pairs of nodes were reachable in only a few hops (Watts and Strogatz, 1998). Jon Kleinberg subsequently showed what properties made these networks efficiently navigable with decentralized search algorithms (Kleinberg, 2000, 2001).

### 2.2. Navigation in information networks

Whereas in Milgram's experiments navigation was conducted in social networks, the focus for navigation research has since shifted to information networks. Information networks imply different characteristics in navigation: in contrast to social networks, navigation is only conducted by a single agent who can more easily explore larger parts of the network (Helic et al., 2013). In terms of decentralized search, this signifies that even though the user conducting the search stays the same over the entire duration, the decision of what link to click is taken independently at each step.

Web navigation can be effectively modeled by computational cognitive models. One of the most prominent models is information foraging (Pirolli and Card, 1999), which models information-seeking behavior based on optimal foraging theory in biology. In this model, *information scent* (Chi et al., 2001) guides users to patches of information. Just as animals are thought to maximize their benefit gained from foraging, information seekers are thought to make optimal use of their resources to gain information. Based on the notion of information scent, the cognitive model of SNIF-ACT has been developed to explain navigation choices in navigating between webpages (Pirolli and Fu, 2003). Another model based on information scent is CoLiDeS (Comprehension-based Linked Model of Deliberate Search) (Kitajima, Blackmon, and Polson, 2000) that explains navigational choices of users within webpages. This model was later extended as CoLiDeS+, which includes the previously visited webpages in the link selection process (Juvina et al., 2005). SNIF-ACT and CoLiDeS are considered complimentary models (Kitajima, Polson, and Blackmon, 2007). Whereas SNIF-ACT models information patches as entire websites, CoLiDeS models the link selection decisions within regions of a webpage. Similar to CoLiDeS, in this paper we study the link selection behavior for different areas of Wikipedia articles.

Information network navigation has also been found useful as an evaluation method for information systems. For medical documents, navigation with decentralized search was found to be comparable to human navigation (Lamprecht et al., 2015b) and was used to point out differences in folksonomy generation algorithms (Helic et al., 2011). Seyerlehner et al. used navigability to examine recommender systems and found top-N collaborative filtering to be inherently poorly navigable (Seyerlehner, Flexer, and Widmer, 2009). Lamprecht et al. later confirmed this finding for the recommendation networks of the Internet Movie Database (IMDb) and suggested to use diversification to make networks more navigable (Lamprecht et al., 2015a).

### 2.3. Navigation on wikipedia

Log data for many Web information systems are chronological—they form trails or traces of user activity. The ultimate and concrete user goals, however, are rarely known directly from log data. In click-trails from logs, goals can occur at any part of a path (if at all) and are not distinguishable from other clicks. To overcome this issue, researchers have resorted to studying click-trails of navigational games such as wikigames. These games (e.g. Wikispeedia^1^ or the Wiki Game^2^) challenge players to reach a predetermined target article only by following links within the body text. Log files from these games equip researchers with concrete start-target scenarios for navigation and allow for a more detailed investigation. Wikipedia logs have been found useful to find semantically similar Wikipedia articles (Singer et al., 2013; West, Pineau, and Precup, 2009) and discover missing links (West, Paranjape, and Leskovec, 2015).

Wikigames have been extensively studied on the Wikispeedia dataset (West et al., 2009). This wikigame is played on the Wikipedia for Schools 2007 selection, a subset of around 4600 articles chosen based on the UK National Curriculum. Players of Wikispeedia have been found very efficient at finding goals (West and Leskovec, 2012b). Paths found by players showed a tendency to navigate (and backtrack Scaria et al., 2014) to a high-degree hub first and then home in on the target based on content similarity (West and Leskovec, 2012b). These characteristics were different from shortest paths or paths found by search algorithms. Surprisingly, simple search algorithms (e.g. based on textual similarity) were even more efficient than humans, proving that no high-level reasoning skills were necessary to find targets (West and Leskovec, 2012a).

### 2.4. Influence of webpage organization

Computational cognitive models such as CoLiDeS model link selection based on the semantic similarity to the target (Kitajima et al., 2000). However, structural knowledge has been shown to have an impact navigation performance (Juvina and van Oostendorp, 2008). In this paper, we compare the influences of textual similarity to the influence of webpage structure.

The organization of a webpage can exert a significant influence on viewing and click decisions. In this paper we study these influences in the context of Western culture and its left-to-right writing direction. Generally, humans are known to be biased by presentation order even in the absence of explicit ranking. In multiple-choice questions, people more frequently select answers located closer to the top (Blunch, 1984; Payne, 1951). The same effects have been identified in cultural markets (Salganik, Dodds, and Watts, 2006) and recommender systems (Lerman and Hogg, 2014). For websearch, users have been found to predominantly focus their attention on the first few results (Buscher, Dumais, and Cutrell, 2010; Craswell et al., 2008; González-Caro and Marcos, 2011; Pan et al., 2007). Eye-tracking has shown that humans scan webpages in an F-shaped pattern (Nielsen, 2006) and generally focus on the top and left areas of webpages (Buscher et al., 2009).

Web users have been found to quickly decide whether a page is worth their interest. Weinreich et al. (2006) report that users stay on most pages only for a short time span and that 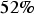 of all visits are shorter than 10 seconds. Web users frequently skim a page at first to determine its relevancy (Liu, White, and Dumais, 2010; Liu et al., 2014). This behavior also shows in the analysis of click locations: in a study, 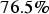 of clicks where made in the area visible without scrolling and 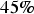 on links located near top left corner (Weinreich et al., 2006).

These findings suggest that users mostly skim content and immediately decide whether to stay or to move on. However, when information needs are not satisfied by the top links, users can adapt and dedicate more attention to all of the search results (Salmerón, Kammerer, and García-Carrión, 2013). This suggests that users attribute certain characteristics to specific parts of webpages but adjust them based on the actual content.

## 3. Datasets

In this work we look at Wikipedia navigation from two distinct vantage points: First, we analyze data from an entire month of all clicks within the English Wikipedia. This allows us to gain insight into unconstrained free-form navigation on the encyclopedia. Second, we study goal-directed Wikipedia navigation based on wikigames, which permits us to inspect complete navigation paths with explicit navigation targets.

### 3.1. Wikipedia clickstream

We investigate free-form click behavior in the English Wikipedia based on a dataset recording all clicks to the Desktop version of the English Wikipedia within the month of February 2015 (Wulczyn and Taraborelli, 2015). Visitors use Wikipedia in many different ways, such as looking up specific facts, trying to learn about a concept, reading articles to pass time, and many more. The Wikipedia clickstream datasets records clicks from all types of visits in aggregate in the form of link click counts for links in articles. As a consequence, the dataset does not reveal any user sessions, and potential navigation sessions or navigation targets cannot be identified. The Wikipedia clickstream therefore allows us to study unconstrained free-form navigation obtained non-reactively (i.e. not subject to potential behavioral changes due to the fact that users knew they were being recorded).

For this work, we only consider clicks between pairs of Wikipedia articles and exclude any external webpages. To allow for a fair comparison with the wikigame dataset described in the following, we restrict our analysis to the same roughly 4600 articles available in Wikispeedia. However, we analyze all clicks from these articles to any article in the English Wikipedia (including all those not contained in the selection). This leaves us with 56,961,992 clicks to study.

### 3.2. Wikigames

For many Web information systems, the log data are chronological and form trails of user activity. The ultimate and concrete user objectives, however, are rarely known relying solely on log data. In click-trails, the exact user goals can be present at arbitrary points in the trails, as there exists no clear distinction of goals within paths.

Navigational games played on Wikipedia (such as Wikispeedia^3^ or the Wiki Game^4^) allow us to circumvent this problem. In these games, the objective is to reach a given target article without using the search function or any external information. These navigation tasks are conducted as follows: Starting from a *start article*, users aim to find a given *target article* by following links in the text. For this work, we use data from *Wikispeedia* (West et al., 2009), a wikigame played on the Wikipedia for Schools 2007 selection. This selection is a subset of the English Wikipedia of around 4600 articles produced for educational purposes by the charity SOS Children. The articles are chosen based on the UK National Curriculum to illustrate educational topics and not necessarily for their link structure and navigability.

In Wikispeedia, players are challenged to play a mission, which by default consists of randomly selected start and target articles. Players can also opt to manually select a start and target article for a mission. Before starting and at any time during the game, players may inspect the full content of the target article (but not its inlinks or related articles). We use the log files of roughly 75,000 wikigames and 475,000 clicks. Table 1 shows a sample of the log files. As a preprocessing step, we resolved all clicks on the back button (logged as “<”) to the corresponding articles. These log files equip us with concrete start-target scenarios for navigation and allow for a more detailed investigation.

**Table 1.** *Sample entries of the Wikispeedia dataset.* The figure shows a part of the log files of successful wikigames. The path column lists the visited pages from the start article (in bold) to the target article (also in bold), separated by semicolons. A < character indicates a back click, which we resolved to the previous page.

hashed IP address	timestamp	path
1d11d305144df277	1233667617	**Wake_Island**;Guam;<;Pacific_Ocean;Peru;Brazil;**Rio_de_Janeiro**
36dabfa133b20e3c	1249525912	**14th_century**;China;Gunpowder;**Fire**
051611353cd98688	1260476499	**Commodore_64**;United_States;India;**New_Delhi**
473d6ac602c2b198	1322605407	**Asteroid**;Jupiter;Roman_mythology;<;<;Comet;Denmark;**Viking**

## 4. Structure of Wikipedia articles

As the first step of our analysis, we study the characteristics of structural organization and hyperlinks. The structural requirements, which Wikipedia articles are expected to follow, are laid out in the encyclopedia's manual of style (Wikipedia, 2016a). Articles should generally start with a *lead section* (or *lead* for short), which is the first section before the table of contents and the first heading. The lead should serve as an easy-to-understand introduction to the article and establish context. Similar to an abstract for a scientific article, the lead should not be divided into any sections (Wikipedia, 2016c).

Articles can optionally contain an *infobox*—a tabular description of the article's most important facts (Wikipedia, 2016b) (e.g. *scientific classification*, *country*, *area* or *date of birth*). In our datasets, infoboxes are present in 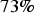 of the 4600 articles from the Wikipedia clickstream and 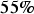 of the articles used in Wikispeedia. Infoboxes generally appear next to the lead section in the top right of an article. Figure 1 gives an example of an article used in Wikispeedia and shows both the lead section and an infobox.

The lead section is followed by the *content* of the article, which is usually divided into a number of sections. Wikipedia guidelines do not specify how to structure the content, and this decision is left to the Wikipedia editors (Wikipedia, 2016a). The content may be followed by appendices (such as references and external links) and footers (such as navigation templates or categories). Due to the fact that the structure of the content part is not regulated and humans have been found to dedicate more attention to the top of lists and webpages (Buscher et al., 2009; Lerman and Hogg, 2014), we will focus our analysis on the structure at the top of Wikipedia articles and its effects on navigation.

### 4.1. Characteristics of lead and infobox

In this section, we explore the characteristics of the structures located near the top of a Wikipedia article. Wikipedia guidelines describe an article's lead section as follows: *The lead serves as an introduction to the article and a summary of its most important contents. […] The opening sentence should provide links to the broader or more elementary topics that are important to the article's topic or place it into the context where it is notable* (Wikipedia, 2016c). Moreover, the number of links in the lead should be restricted to what is required (Wikipedia, 2016d). These guidelines suggest the presence of more general links in the lead sections of articles.

*Approach*. To assess whether the top of an article contains more general links, we compare the generality of links in the lead, the infoboxes and the remainder of the article. To measure generality, we make use of the usage frequency, which has been identified as a good proxy measure for generality (Benz et al., 2011). We use the following three measures:

- *Indegree*: The indegree of an article is the number of links pointing to it from within Wikipedia. Indegree measures the *navigational quality* of a node as well as *generality* (Gabrilovich and Markovitch, 2009, p. 450). Indegree captures the generality as viewed by Wikipedia editors, who placed these links.
- *View count*: We use the number of views ^5^ that Wikipedia articles received in February 2015 (the same time that the Wikipedia clickstream was collected). The number of times an article is visited measures the popularity and generality as seen by Wikipedia visitors.
- *Search query n-gram frequency (n-grams)*: We measure the familiarity and generality of a term in the active vocabulary of users of a websearch engine by the number of occurrences of article titles in search queries to Microsoft Bing (from the Microsoft Web N-gram corpus Wang et al., 2010).

**Figure 2.**
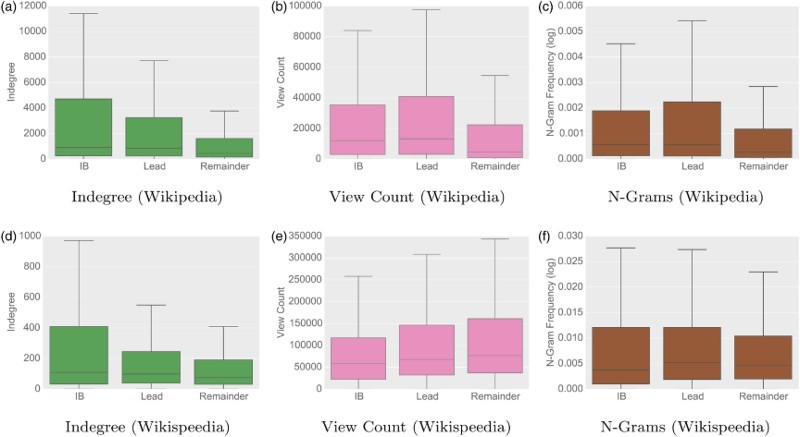
*Indegree, view count and search query n-gram frequency for lead, infoboxes and the remainder of articles.* The figure shows the values for the link targets in these sections (outliers are excluded for the sake of clarity). Articles linked in the lead and infobox have a higher indegree, which holds for both Wikipedia and Wikispeedia. For Wikipedia we also find that articles linked in the lead have a higher view count and higher n-gram frequencies. These results show that links towards the top of an article tend to lead to more general articles. (a) Indegree (Wikipedia), (b) View Count (Wikipedia), (c) N-Grams (Wikipedia), (d) Indegree (Wikispeedia), (e) View Count (Wikispeedia) and (f) N-Grams (Wikispeedia).

*Results*. Articles linked in the lead and infobox mostly lead to pages with a higher generality (cf. Figure 2). For the Wikipedia clickstream, links in the lead sections and infoboxes indeed lead to articles with a higher generality in all of the three measures. This confirms our hypothesis that these sections contain links to more general articles.

For the articles used in Wikispeedia, we find that links in the lead and infobox also lead to articles with a higher indegree. However, this does not hold true in terms of view count and only for the lead section in terms of search query n-gram frequency. One possible explanation for this is the limited number of possible link targets in Wikispeedia, where links are restricted to articles within the subset of Wikipedia for schools. This may introduce a bias, since a large number of the more specific articles are not available as link targets, increasing the generality of links outside the lead and infobox. However, when considering the network-intrinsic measure of indegree, links in the lead and infobox do lead to more general articles. These results show that links towards the top of an article tend to lead to more general articles.

### 4.2. Links in Wikipedia articles

Hyperlinks in Wikipedia articles are meant to establish context, help to understand the content better and provide references to more background information (Wikipedia, 2016e). As a general rule, *a link should appear only once in an article, but if helpful for readers, may be repeated in infoboxes, tables [··· ] and at the first occurrence after the lead* (Wikipedia, 2016d). Specifically, the article should be complete even without reading the infobox (Wikipedia, 2016b).

This indicates that links can occur multiple times within the same article. And indeed, for the Wikipedia clickstream, 49.0% of link targets are linked multiple times within the same article, and these ambiguous link targets received 75.8% of all clicks in the dataset. For Wikispeedia, 28.5% of all link targets on an article are linked multiple times and received 43.6% of all clicks.

To the best of our knowledge, there exist no public Wikipedia datasets which contain the information of the exact position of clicked links. We therefore measure the influences of lead and infobox by comparing link selection models, as described in the following section.

## 5. Influences on aggregated Wikipedia navigation

To establish the influence of article structure on navigation choices on Wikipedia, we now turn our attention to link selection models. These models allow us to obtain the degree of influence of article structure, which we then compare to other potential influences on navigation. We study a range of influences on aggregated Wikipedia click data in the form of (i) the clickstream (which is the aggregate of many sessions) and (ii) the aggregate of all clicks from the Wikispeedia logs. To this end, we combine all clicks from all Wikispeedia games in Wikispeedia and count the clicks on each link for each article. This aggregation therefore excludes all information about game paths and allows us to compare both datasets in the same form.

### 5.1. Influencing factors

We investigate the following influences on navigation:

*Article structure*: Wikipedia articles follow specific guidelines that lead to structural regularities. We examine the influence of
  - *Lead*: The first section of an article
  - *Infobox*: An optional tabular description of the article's main facts
*Generality*: We investigate three factors for the generality of articles (cf. Section 4.1):
  - *Indegree*: generality as seen by Wikipedia editors
  - *View count*: generality and popularity as seen by Wikipedia visitors
  - *Search query n-gram frequency (n-grams)*: generality and familiarity as seen by search engine users
*TF-IDF similarity to the target*: We measure the cosine term frequency-inverse document frequency (TF-IDF) similarity between an article and the target article of goal-directed navigation (only evaluated for wikigames, where targets are explicitly known).

### 5.2. Link selection models

As a large share of articles on Wikipedia includes repeated links to the same target (cf. Section 4.2), we cannot resort to simply counting the number of clicks in each section. Therefore, to model link selection, we assign each link a probability based on the influencing factors. We then evaluate the models and compare them to the clicks made by users. This approach implies a memoryless navigation process, which has been found to be a good fit for human navigation of Wikipedia (Singer et al., 2014).

*Approach*. We investigate the influence of infoboxes and the lead sections as follows. First, we set a probability *p* for the section (e.g. *p*=0.6). We then distribute this probability uniformly over all links within the section and remaining probability of 1−*p* (e.g. 0.4) over the links in the remainder of the article. We investigate values for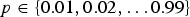.

For indegree, view count and search query n-grams, we set the link selection probability to be proportional to the value of these factors for the link target. For example for indegree, we first compute the sum *s* of the indegrees 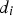 of all link targets *i* reachable from a given article and then assigned each link target *i* a probability of 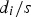.

To model the influence of textual similarity to the navigation target, we model the TF-IDF cosine similarity between the text of the current and the target article and assign each link a probability proportional to the TF-IDF similarity of the link target to the navigation target. This is possible only for Wikispeedia, where navigation targets are explicitly known.

Finally, we use a uniform model as the baseline, where we assign each link *l* a weight of 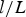, where *L* is the number of links in the article (and hence assign each link a uniform click probability).

*Kullback–Leibler (KL) divergence*. To compare the models to the ground truth (i.e. the clicks made by users, we use the KL divergence. Let *p* and *q* be two discrete probability distributions on 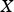. The KL divergence of *q* from *p* is then(1) 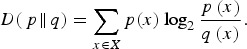
The KL divergence measures the distance of the distribution *q* from *p* and states the expected number of additional bits needed to code samples from *p* when an optimal code for *q* is used instead. This gives us a measure for how well a distribution can be used to approximate another. We use a Laplace smoothing of 0.0001 for all values to avoid any problems with zero entries.

*Model evaluation*. We evaluate the effects of the influencing factors as follows:

1. We count the number of all user clicks going away from an article of the dataset. We denote these as the *outclicks*.
2. We use the influence model to compute the link selection probability of each link in an article. We then multiply these probabilities by the number of *outclicks* registered from that article. For example, for a link with selection probability of 0.2 for a model and 8 registered *outclicks* on the article, this would result in a value of 1.6 for the target article.
3. We count the sum of values received this way by each article from any other article. We denote these as the *inclicks* of articles.
4. Finally, we normalize the *inclicks* over all articles. We then compare this distribution of *inclicks* to articles to the normalized ground truth from the dataset (i.e. the number of times articles were clicked by users) by computing the KL divergence for substituting the ground truth with the result of the link selection models.

### 5.3. Results

**Figure 3.**
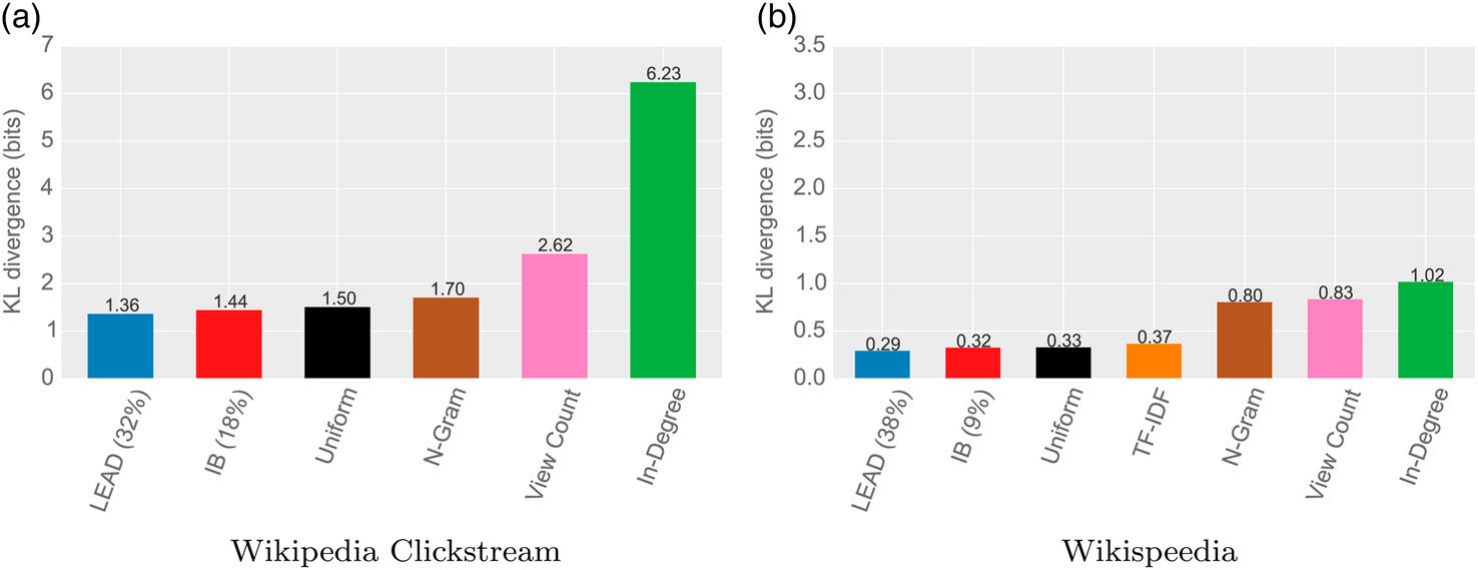
*KL divergences for link selection models when substituting for the ground truth distribution of clicks.* The figure shows the KL divergence when substituting the distribution of clicks to articles with a range of link selection models. For the models regarding infobox (IB) and lead, only the models with the smallest KL divergence are shown. Models based on article structure explain navigation choices best, followed by the uniform model. Generality-based models are not a good fit. (a) Wikipedia Clickstream and (b) Wikispeedia.

Figure 3 shows the results of the comparison of KL divergences of the distributions when substituting for the ground-truth distributions.

*Article structure best explains navigation choices*. The models with the lowest KL divergence to the ground distribution are the ones placing more importance on lead or infobox. The resulting models also represent the importance of these sections: Wikipedia is best fit with 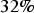 of all clicks made in the lead or 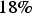 in the infobox. This is substantially more than the link counts would suggest: For Wikipedia, the lead contained 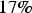 and the infobox contained 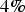 of the links on articles. This indicates that these section are considerably more important for users than the number of present links would suggest. The same holds true for Wikispeedia, where the infobox (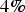 of all links) and the lead (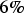 of links) are again better fitted with higher importance weights.

*Generality is not a good explanation for navigation choices*. Indegree, view count and search query n-grams all lead to distributions with large differences to the ground truth. A tendency to click on more general articles is therefore not a good fit for explaining the aggregate of all clicks.

*Selecting links uniformly is a good fit*. The baseline model, selecting links uniformly at random across the entire article, is able to fit navigation choices comparatively well. This indicates that navigation uniformly at random (such as the one used to compute PageRank) can serve as a useful model for explaining the aggregate of all clicks to Wikipedia.

*TF-IDF similarity to the target is a fairly good fit*. The TF-IDF similarity to the target leads to a larger divergence than the uniform model, but still considerably better than the generality models. This suggests that textual similarity plays an important role in the goal-directed navigation of Wikigames.

*Both datasets show similar characteristics*. The Wikipedia clickstream and the Wikispeedia datasets we investigated consist of data from different navigational situations: While the Wikispeedia dataset is restricted to focused, goal-directed navigation, the Wikipedia clickstream dataset comprises a large range of forms of navigation. Despite this difference, the navigational influences for both datasets are very much alike. For both datasets, the order in goodness of fit for the resulting models is exactly the same. This indicates that, in their aggregate form, Wikispeedia is subject to the same influences as the Wikipedia in its entirety. The most notable difference is the larger KL divergence for Wikispeedia for N-Gram and View Count. However, this is likely due to the restriction to a subset of articles (cf. Section 4.1).

Overall, article structure best explains Wikipedia navigation when aggregating over all clicks and navigation scenarios. However, we see that TF-IDF similarity to the target is also a fairly good explanation for navigational choices in Wikispeedia. In the light of these findings, we now investigate the influence of structure in a step-by-step analysis of goal-directed navigation.

## 6. Influences on goal-directed Wikipedia navigation

We now shift our analysis to goal-directed Wikipedia navigation in the form of wikigames. Wikigames challenge users to retrieve articles by following links in articles without using any external help or the search function. As such, they are examples of goal-directed navigation: starting from a given article, the aim is to find a target article with as few clicks as possible. Log files from these games have the inherent advantage of explicitly specifying the navigation target at all times, therefore permitting us to examine navigation step-by-step.

Two navigational phases have been identified for wikigames: (i) an initial zoom-out phase where players navigate to high-degree nodes, and (ii) a final home-in phase to the target, in which players navigate based on textual similarity to the target (West and Leskovec, 2012b). In what follows, we aim to identify the impact of article structure on the goal-directed navigation of wikigames.

*Approach*. To quantify the impact of different influences on goal-directed navigation, we conduct the simulation and analysis of link selection models as described in Section 5.2. However, we now evaluate the models step by step. To study both the initial and the final stage of the game, we restrict our analysis to successful games, for which users were able to find the target article. These make up around two thirds of the dataset (around 50,000 out of 75,000 games). Our approach was as follows:

1. We split up all games by (a) shortest possible solution and (b) game length (i.e. clicks it took the user to find the goal). This partitions games into classes, e.g. all paths of length eight for games with a shortest solution of three clicks.
2. For each class of games, we evaluate the link selection models separately for every step. We restrict the ground truth clicks to the ones performed in one step of the class and thus model each step in every partition class on its own.
3. We compare the models to the ground-truth models for each step by computing the KL divergence.

This approach leaves us with models for every step in every class of the partitioned games and allows us to compare the corresponding best fits.

**Figure 4.**
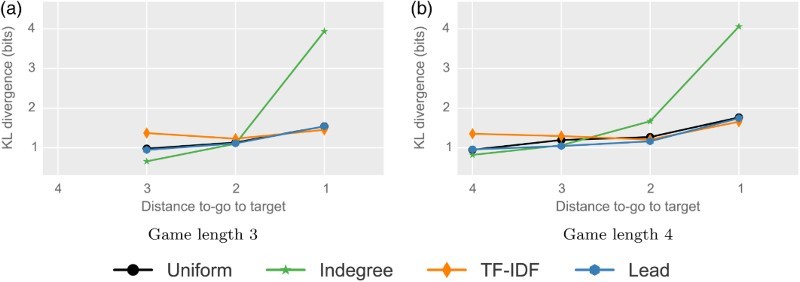
*Comparison of navigation strategies.* The figures show the KL divergences of the link selection models to the user clicks for the top three models and the random baseline for games with a shortest possible solution of three clicks for which users found solutions of (a) three and (b) four clicks. In the beginning of games, the indegree model performs best. Towards the end, the TF-IDF model has the lowest KL divergence, while in between these phases, article structure is a good fit. This shows that the influence of article structure is notable in all but the first and last clicks. (a) Game length 3 and (b) Game length 4.

*Results*. Figure 4 shows the results of the application of the link selection models to two classes of (short) games and displays the three best-performing models, the uniform model and their resulting KL divergences to the user click distribution. The results show that in the beginning of games, the indegree model performs best. Towards the end, the TF-IDF model has the lowest KL divergence. These correspond to the zoom-out and the home-in phases in line with the work of West and Leskovec (2012b). All other steps (in the central stages of the games), however, are best modeled based on article structure.

**Figure 5.**
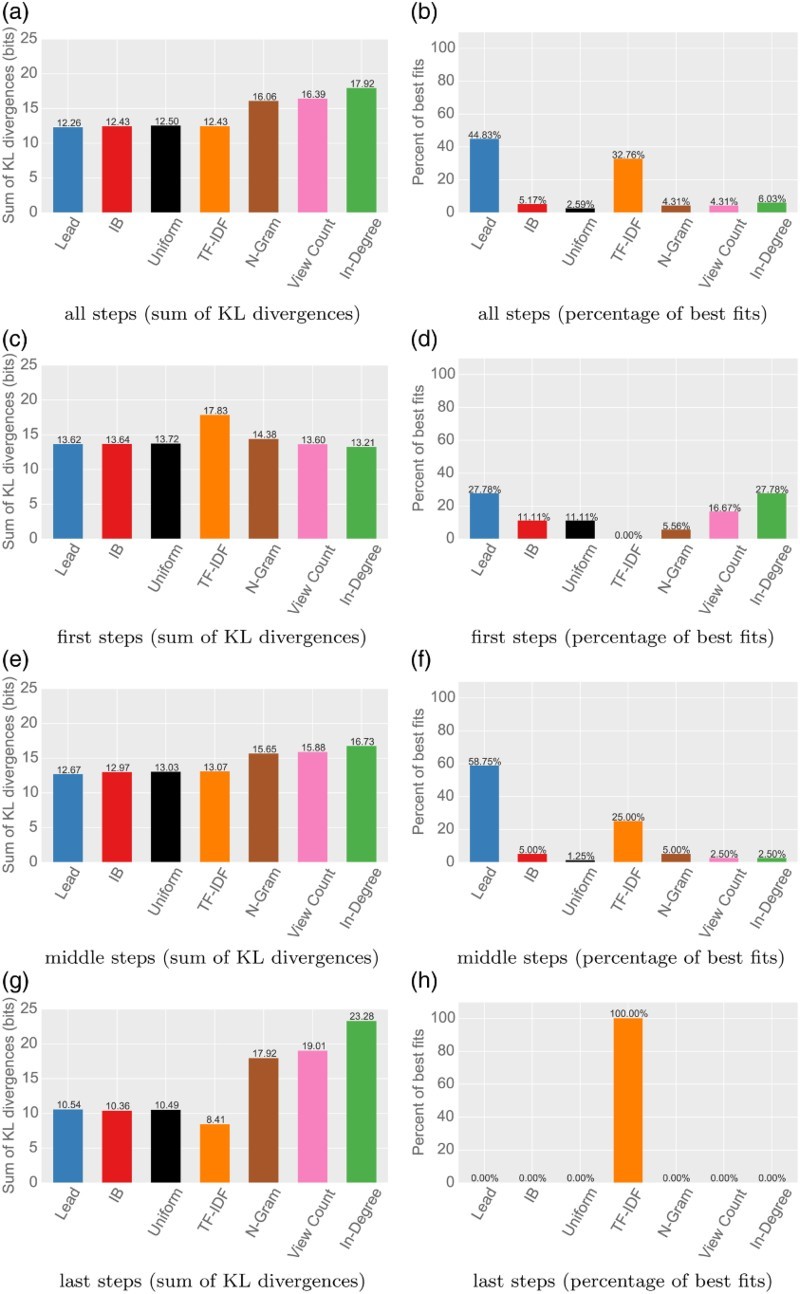
*Step-by-step analysis of successful games.* The figure shows the analysis of successful navigation paths. Games were partitioned into classes by (a) shortest possible solution (3, 4 and 5 clicks) and (b) game length (i.e. clicks it took the user to solve it, maximum 10). E.g. all paths of length eight for games with a shortest solution of three clicks. We then evaluated click models to every step in these partitioned games, leaving us with 146 models. The left column shows the sum of KL divergences for these models. The right column shows the fraction of stepwise results for which a model was the best fit. Article structure is able to explain navigational choices best in all except the first and last steps. (a) all steps (sum of KL divergences) (b) all steps (percentage of best fits), (c) first steps (sum of KL divergences), (d) first steps (percentage of best fits), (e) middle steps (sum of KL divergences), (f) middle steps (percentage of best fits), (g) last steps (sum of KL divergences) and (h) last steps (percentage of best fits).

We now analyze all successful games with shortest possible solutions of 3, 4 and 5 clicks and user paths with up to 10 clicks, which comprises a total of 23,717 games and 76,874 clicks. Figure 5 shows the results of this step-by-step analysis of a total of 146 link selection models (one for every step in every class of games).

*Generality explains the initial phase*. The first steps in games are best explained by generality-based measures (indegree, view count and n-grams), which together make up half of the best fits for all first steps. This confirms that in the initial phase of navigation, users tend to select a link leading to a general and high-degree article—the zoom-out phase. A likely explanation for this phase would be that users start out from an unfamiliar article and try to get to a landmark serving as a point of orientation.

*TF-IDF similarity explains the endgame*. The last clicks in games are overwhelmingly best fit by TF-IDF similarity models. This again confirms previous work, which found that users tend to navigate by textual similarity in the endgame.

*Overall, article structure best fits the largest fraction of steps*. For combination of the models for all steps, the lead model has the smallest KL divergence to the distribution of user clicks. Together, the lead and the infobox models are the best fit for half of the steps in the games, followed by the TF-IDF similarity to the target. When we exclude the initial and final phases of games, article-based models best fit around 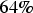 of all steps. This indicates that in all except the initial and final phases of games, the bias to article structure is actually substantially stronger than biases to generality or TF-IDF similarity.

*Influence of game design*. The step-by-step analysis of navigation paths shows that the first and last steps are substantially different from the remainder of clicks. This is possibly due to the influence of game design on these paths: The first click frequently serves to get to a landmark article serving as a point of orientation. If we compare this to regular Web use, where powerful search engines are available, it appears less likely that the first step would be necessary in a setting where users find it easy to start information retrieval with a query to a search engine.

Regarding the last step, we notice that links in the Wikipedia for Schools selection of articles (on which Wikispeedia is based) occur proportionally to the TF-IDF similarity between articles (West and Leskovec, 2012b). It therefore appears likely that this causes the last link in navigation paths to be best explainable by TF-IDF similarity.

If we exclude the first and last steps and examine the combination of the remainder of steps, the models are in the exact same order as the aggregate of all steps for the Wikipedia clickstream as well as the aggregate of all clicks in successful and unsuccessful games for Wikispeedia (cf. Figure 3). We therefore argue that the first and last steps are outliers in a sense—the reasons for the click decision in these steps could well be due to the way the wikigame is set up and not reveal the true navigational needs of users. As such, we believe that a bias towards article structure is able to explain the largest fraction of navigational decisions in goal-directed navigation.

In summary, these results suggest that the characteristic first and last steps of wikigames are subject to different conditions. Outside these two phases, however, navigational choices can best be modeled by focusing on the article structure as well as the textual similarity to the navigation target.

## 7. Discussion

In this paper we studied human navigation of Wikipedia by investigating logs of click data from two perspectives: (i) a combination of many usage scenarios (the Wikipedia clickstream) and (ii) focused goal-directed navigation (Wikispeedia). We investigated the following research question:

*Research question*: To what extent does article structure affect Wikipedia navigation?

We addressed this question by looking at influences on Wikipedia navigation from the perspectives of aggregated navigation and of goal-directed navigation.

*Aggregated navigation*. The aggregated navigation data combine measurements from a large number of visits to Wikipedia. For these data, our results show that the structure of Wikipedia articles has a substantial influence on human navigation. We compared a range of potential navigational influences and found that, overall, article structure has a larger influence on navigational choices than generality. The influence of TF-IDF similarity was substantial, but slightly less strong than the influence of article structure. This confirms previous work that has found both semantic and structural knowledge to influence navigation performance (Juvina and van Oostendorp, 2008). Our analysis showed that links occurring in the lead section or the infobox play an especially important role for navigation. The best-fitting models for the lead and infobox placed a weight on these sections that was substantially higher than the number of links in them would lead to expect. These results hold true for the aggregate of all clicks recorded for the Wikipedia clickstream as well as for wikigames. In fact, the order of best fits of the navigational influences for wikigames exactly matches the one for Wikipedia. This suggests that the aggregate clicks of goal-directed wikigames are very similar in nature to free-form Wikipedia navigation.

*Goal-directed navigation*. A more detailed analysis of goal-directed navigation in the form of wikigames showed that the navigational decisions in the navigation paths are subject to very similar influences as the aggregated clicks. If we exclude the first and last steps—which have been found to be special cases and might well be caused by the specific setup of the games—the influences are ranked in exactly the same order as for the aggregate of all clicks. Our analysis suggests that the elaborate structure of Wikipedia articles, with a lead-section at the start of the article linking to broader, better known concepts, helps users in the navigation task by making high-degree hubs easier to find. Thus, even if a user were completely lost, clicking randomly on the links appearing on their screen (which tend to be the links in the article lead and infobox), is likely to bring them to a familiar, broader concept, which the user can then use as a point of orientation in the information space. In effect, it is the structure of information in the article that guides navigation, and not necessarily to the network structure.

*Comparison of free-form and goal-directed navigation*. The Wikipedia clickstream covers all possible usage scenarios: looking up facts, learning about concepts, reading to pass time, and many more. The Wikispeedia dataset is more specific and is limited to goal-directed navigational games, which also make up an unknown fraction of the clicks in the clickstream. As such, we expected the navigational influences to differ between the datasets. However, our results have shown that the aggregated clicks from Wikispeedia led to the same order of influence model results as the Wikipedia clickstream. This could be due to two reasons: It could mean that a large fraction of the clicks in the clickstream stems from goal-directed navigation and therefore the link selection models hold true for them as well. This would imply a confirmation of our results on a second dataset. If, on the other hand, the majority of clicks from the clickstream stems from navigation scenarios other than goal-directed navigation, this would mean that the navigational influence models we presented are valid for the combined clicks of other scenarios as well. This in turn would imply that our models generalize to the aggregate of all Wikipedia usage scenarios. Due to the limited availability of Wikipedia click logs, we are unable to answer investigate this further at this point. However, should more detailed log data of Wikipedia become available, it would be fruitful to expand our work to it.

*Limitations and future work*. The setup as of Wikispeedia as a game as well as the reactive approach to collecting this data (users knew that their log data would be used for evaluations) could potentially introduce biases. However, our step-by-step analysis has shown that while the first and last clicks in Wikispeedia show different characteristics than the Wikipedia clickstream, we could find no substantial difference for the remainder of clicks. As such, we believe that wikigames, despite of their game setup, can provide us with valuable insight into real-world navigation behavior in large information networks. As mentioned, it would certainly be worth repeating this analysis on navigation paths collected from real-life navigation behavior on Wikipedia. This would also open up the opportunity to compare the navigation behavior of different types of users and different usage scenarios, which would deepen our understanding of the navigation dynamics on Wikipedia.

The structure-based link selection models were based on assigning a section of the article a higher weight. The simplicity of this approach raises the questions if more detailed position models could be able to even better explain click selection strategy. A possible extension of this work would be a combination of click models for several sections or influences. Another follow-up study could be to use two separate versions of Wikipedia articles restructured according to different information architectures and observe users navigate on them.

The step-by-step analysis of navigational influences could be used to assess performance levels of users. For example, the navigational influences at the current step could be compared to the typical influences on well-performing users at that step. This could be valuable to, for example, intelligent systems relying on data mining performed by its users. A first step in this direction could be the analysis of unsuccessful wikigames.

Finally, our analysis focuses on the desktop view of Wikipedia for both datasets. In future work, it would be interesting to repeat this analysis for the Wikipedia view for mobile devices, which display Wikipedia in a different design (e.g. infoboxes appear before the lead section instead of next to it as in the desktop view).

## 8. Conclusion

We have shown that the decentralized organization of Wikipedia leads to elaborate structure in the articles of the encyclopedia, greatly facilitating navigation. This structure and regularity substantially helps user to navigate the information network. Our results clearly demonstrate the navigational importance of links in terms of their position in an article. We have found evidence that a large share of clicks to articles in the English Wikipedia are to links in the lead and the infobox, which in turn suggests that the majority of visitors focus their attention on these sections. This finding is relevant for the shaping of Wikipedia policies and suggests that the upper part of articles should receive the most attention, e.g. in terms of fact checking or monitoring for vandalism. Our results also suggest that attention needs to be paid to edits that change an article only by moving text to another location: given our results, it seems likely that placing sections containing criticism before other content can drastically alter a user's impression of an article without even changing any of the words in it.

Our work also helps to understand the impact of the partitioning of Wikipedia articles into sections. Information from the lead section or the infobox is frequently used in external sites (e.g. by search engines). Our results show that this in fact frequently matches what users actually see when they look at an article itself. In terms of Wikipedia administration, special attention should therefore be paid to the neutrality and balance of the lead section.

## Notes

1 
www.wikispeedia.net

2 
www.thewikigame.com

3 
www.wikispeedia.net

4 
www.thewikigame.com

5 Retrieved from http://stats.grok.se
